# Novel Developments in Acute Kidney Injury

**DOI:** 10.1155/2016/2756204

**Published:** 2016-08-10

**Authors:** Jeremiah R. Brown, Peter A. McCullough, Michael E. Matheny

**Affiliations:** ^1^The Dartmouth Institute for Health Policy and Clinical Practice, Geisel School of Medicine, HB 7505, 1 Medical Center Drive, Lebanon, NH 03756, USA; ^2^Department of Medicine, Dartmouth-Hitchcock Medical Center, (DHMC), HB 7505, 1 Medical Center Drive, Lebanon, NH 03756, USA; ^3^Department of Community and Family Medicine, Dartmouth-Hitchcock Medical Center (DHMC), HB 7505, 1 Medical Center Drive, Lebanon, NH 03756, USA; ^4^Baylor Heart and Vascular Institute, 621 North Hall Street, H030, Dallas, TX 75226, USA; ^5^Geriatrics Research Education & Clinical Center (GRECC), Tennessee Valley Healthcare System (TVHS), Veteran's Health Administration, USA; ^6^Division of General Internal Medicine, Department of Medicine, Vanderbilt University School of Medicine, Nashville, TN 37232, USA; ^7^Department of Biomedical Informatics, Vanderbilt University School of Medicine, Nashville, TN 37232, USA; ^8^Department of Biostatistics, Vanderbilt University School of Medicine, Nashville, TN 37232, USA

Over the past decade, acute kidney injury (AKI) has emerged rapidly in the scientific literature and clinical awareness. There have been great strides in understanding the pathophysiology and adverse outcomes associated with AKI. In this special issue on acute kidney injury (AKI), contributing authors discuss novel clinical and experimental research emerging in the field of AKI.

*Clinical*. Several contributions provide evaluations of AKI in clinical practice. J. Brown et al. start off the special issue on AKI by reviewing the trends in AKI related hospitalization in the United States of America over the past decade. O. E. Ibrahim et al. evaluated the association of elevated serum creatine kinase (>2500 U/mL) as a marker for rhabdomyolysis and AKI after high risk adult cardiac surgery. A. Kwizera et al. explore the association of intermittent hemodialysis for treating AKI in an African Intensive Care Unit. 

*Basic Science*. Likewise, novel experiments and investigations to better our understanding of AKI in animal models provide important insights into future AKI exploration. C. Jiang et al. report on a novel active ingredient of a Chinese herb known to be kidney protective called Tanshinone IIA showing a reduction in folic-acid induced kidney dysfunction through attenuating renal fibrosis after acute kidney injury in a mouse model through inhibition of fibrocytes recruitment. S. M. Fernandes et al. investigate the association between iodinated contrast and renal function in rats with chronic kidney disease and chronic hyperglycemia.

Several experiments focused on the issue of renal ischemia-reperfusion injury in animal models. E. C. Costalonga et al. examined whether valproic acid could prevent renal ischemia-reperfusion injury and subsequent AKI in male Wistar rats. S. Ozbilgin et al. explored whether ischemic preconditioning could prevent reperfusion injury in diabetic rats. Lastly, D. Wen et al. in an in vivo model discuss the role of a DNA binding 1 inhibitor during renal ischemia-reperfusion injury.

*Review of the Literature.* Two reviews provide a critical appraisal of the literature on the role of cisplatin nephrotoxicity and a technology evaluation on the role of renal magnetic resonance imaging in patients with AKI. G. Oh et al. provide a review on cisplatin nephrotoxicity operating through oxidative stress and inflammation and by the reduction of the intracellular NAD^+^/NADH ratio. H. Y. Zhou et al. discuss the role of functional renal magnetic resonance imaging as an assessment tool to evaluate renal morphology and function in the management of AKI.

*Jeremiah R. Brown* *Peter A. McCullough* *Michael E. Matheny*

